# The Role of Lysyl Oxidase Enzymes in Cardiac Function and Remodeling

**DOI:** 10.3390/cells8121483

**Published:** 2019-11-21

**Authors:** Cristina Rodríguez, José Martínez-González

**Affiliations:** 1 Institut de Recerca Hospital de la Santa Creu i Sant Pau-Programa ICCC, 08025 Barcelona, Spain; 2 Instituto de Investigación Biomédica Sant Pau (IIB-Sant Pau), 08041 Barcelona, Spain; 3 CIBER de Enfermedades Cardiovasculares, Instituto de Salud Carlos III, 28029 Madrid, Spain; 4 Instituto de Investigaciones Biomédicas de Barcelona (IIBB-CSIC), 08036 Barcelona, Spain

**Keywords:** lysyl oxidases, cardiac function, myocardial hypertrophy, extracellular matrix remodeling

## Abstract

Lysyl oxidase (LOX) proteins comprise a family of five copper-dependent enzymes (LOX and four LOX-like isoenzymes (LOXL1–4)) critical for extracellular matrix (ECM) homeostasis and remodeling. The primary role of LOX enzymes is to oxidize lysyl and hydroxylysyl residues from collagen and elastin chains into highly reactive aldehydes, which spontaneously react with surrounding amino groups and other aldehydes to form inter- and intra-catenary covalent cross-linkages. Therefore, they are essential for the synthesis of a mature ECM and assure matrix integrity. ECM modulates cellular phenotype and function, and strikingly influences the mechanical properties of tissues. This explains the critical role of these enzymes in tissue homeostasis, and in tissue repair and remodeling. Cardiac ECM is mainly composed of fibrillar collagens which form a complex network that provides structural and biochemical support to cardiac cells and regulates cell signaling pathways. It is now becoming apparent that cardiac performance is affected by the structure and composition of the ECM and that any disturbance of the ECM contributes to cardiac disease progression. This review article compiles the major findings on the contribution of the LOX family to the development and progression of myocardial disorders.

## 1. Introduction

Lysyl oxidase (LOX) proteins comprise a family of copper-dependent enzymes which governs extracellular matrix (ECM) homeostasis and remodeling. Five highly homologous LOX proteins have been identified in mammals: LOX and four LOX-like isoenzymes (LOXL1–4). The primary role of LOX enzymes is to oxidize lysyl and hydroxylysyl residues from collagen and elastin chains into highly reactive aldehydes, which spontaneously react with surrounding amino groups and other aldehydes to form inter- and intra-catenary covalent cross-linkages [1,2].

In the adult mammalian heart, an intricate network of ECM proteins provides a scaffold for the cellular components and participates in the transmission of contractile forces. Studies in both humans and in animal models have evidenced that the abnormal expression/activity of LOX/LOXLs isoenzymes is linked to cardiovascular diseases [1,3,4]. Excessive myocardial collagen deposition and cross-linking (CCL), a process conditioned by LOX enzymes, contributes to cardiac fibrosis and determines left ventricular (LV) stiffness and dysfunction [5,6,7]. Essentially, fibrosis is characteristic of all forms of heart disease, and most of the pathophysiological stimuli that trigger cardiac fibrosis modulate LOX/LOXLs, playing a critical role in the process [8,9,10]. Beyond CCL, additional biological functions have been reported for LOX/LOXLs [1,2]. Further, genes encoding LOX enzymes are part of the mechanoresponsive gene programs responsible for the mechanical regulation of gene expression in cardiac myocytes and fibroblasts [11]. Thus, LOX/LOXLs contribute to cardiac fibrosis, but are also active players involved in the cellular mechanisms underlying cardiac dysfunction and disease progression.

In the last years, multiple studies have contributed to the understanding of the pathophysiological role of the LOX family in human diseases and, in particular, in the cardiovascular system. The involvement of this family on vascular diseases has been recently reviewed [12]. Further, LOX/LOXLs participate in a variety of processes affecting the heart, from those associated to post-myocardial infarction (MI) cardiac remodeling, cardiac hypertrophy triggered by pressure or volume overload, heart failure (HF) with preserved ejection fraction (HFPEF) associated with obesity and metabolic syndrome or atrial fibrillation. The strong impact of the alteration of LOX/LOXLs on CCL and heart function supports the interest of these family of enzymes as pharmacological targets to improve cardiac remodeling and slow the progression to HF. In this review article we focus on those findings that support the fundamental involvement of the LOX family in the mechanisms underlying cardiac damage and repair.

## 2. The LOX Family of ECM-Modifying Enzymes

**Figure 1:**
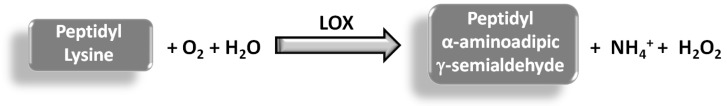
The reaction catalyzed by LOX. LOX activity allows the oxidation of peptidyl lysine residues in collagen and elastin chains to the corresponding peptidyl aldehydes.

LOX is an extracellular enzyme which governs the post-translational modification of ECM collagen and elastin. LOX catalyzes the oxidative deamination of specific ε-amino groups of lysine and hydroxylysine residues in collagen and elastin. This leads to the generation of highly reactive peptidyl α-aminoadipic γ-semialdehydes and the release of hydrogen peroxide as by-product [1,2,3] (Figure 1). This reaction allows the covalent cross-linking of collagen and elastin, which ensures the appropriate tensile strength and elastic properties of connective tissues.

**Figure 2:**
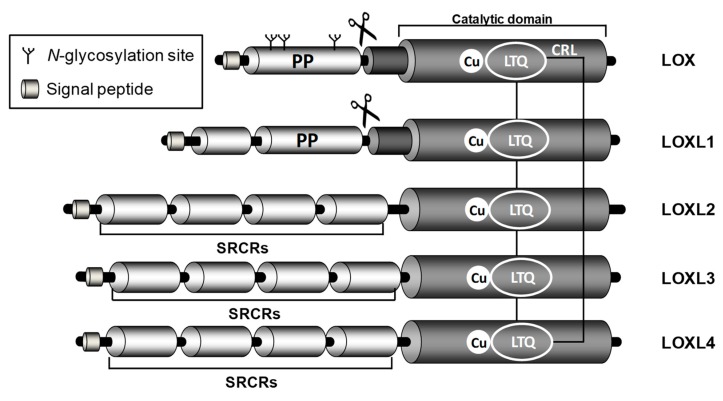
Structure of human LOX isoenzymes. The scheme evidences the high homology of LOX/LOXs isoenzymes in the C-terminal region, formed by the conserved catalytic domain which encompasses the copper binding site and the lysyl tyrosyl quinone (LTQ) cofactor region. The C-terminal region also includes the cytokine receptor-like (CRL) domain. LOX and LOXL1 propeptide sequences (PP) and the site recognized by BMP1 (symbolized by scissors), which allows the cleavage of these isoenzymes and the release of the active forms, are shown. The *N*-glycosylation sites at the LOX-PP region are outlined. LOXLs isoenzymes contain four scavenger receptors cysteine-rich (SRCR) domains probably involved in protein–protein interaction.

LOX is the archetypal member of this family that comprises five closely related copper-dependent enzymes: LOX and four LOX-like isoenzymes (LOXLs: LOXL1, LOXL2, LOXL3, and LOXL4) [13]. Although they are product of distinct genes, all of them show a highly conserved catalytic domain and diverge in the rest of the sequence. Indeed, this family possesses a conserved carboxyl-terminal (C-terminal) amine oxidase catalytic domain, which comprises a His-X-His-X-His copper-binding motif and the lysine tyrosyl quinone (LTQ) cofactor. By contrast, the amino-terminal (N-terminal) domains of these enzymes are structurally unrelated and their biological role is largely unknown. Based on the primary structure of their N-termini, the family can be divided in two subgroups: LOX and LOXL1, which contain a highly basic pro-peptide (PP) sequence, and LOXL2, LOXL3 and LOXL4 which possess four tandem repeats of the scavenger receptor cysteine-rich (SRCR) domains that could mediate protein-protein interactions [14] (Figure 2). The high homology found in the catalytic domain suggests that all of these isoenzymes would have similar substrate requirements and catalytic properties. However, the expression pattern of LOX and LOXLs differ among different tissues, and the dissimilar phenotypes shown by specific knockout animal models indicate that these isoenzymes could play different pathophysiological roles.

**Figure 3:**
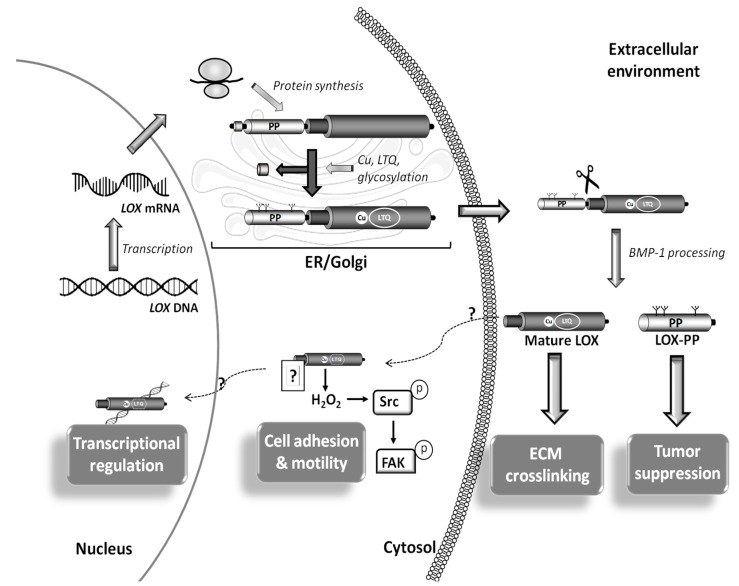
Synthesis and processing of LOX. *LOX* is transcribed and transduced leading to the production of the pre-proLOX form, a pre-proenzyme which is post-translationally modified in endoplasmic reticulum (ER) and Golgi to generate the LOX proenzyme. This multistep process involves (i) cleavage of signal peptide, (ii) incorporation of copper, (iii) formation of the lysyl tyrosyl quinone (LTQ) cofactor and (iv) glycosylation of the LOX propeptide region (LOX-PP). Then this inactive precursor is released into the extracellular space, where it is proteolyzed by procollagen C-proteinases (mainly bone morphogenetic protein 1; BMP1) producing the mature catalytic LOX form, which promotes extracellular matrix (ECM) maturation, and its pro-peptide, which is responsible of the tumor suppressor properties of LOX among others effects. Intracellular forms of mature LOX have also been detected in cytosol and nuclei. In cancer cells, cytosolic active LOX forms control cell adhesion and motility through the H_2_O_2_-dependent activation of Src-kinase and the subsequent phosphorylation of focal adhesion kinase (FAK). Likewise, nuclear LOX modulates chromatin structure affecting gene expression.

LOX and LOXL-1 are synthesized and secreted into the extracellular environment in the form of inactive zymogens, which are subsequently cleaved by bone morphogenetic protein-1 (BMP-1) and other procollagen C-proteinases. This proteolytic processing releases the catalytically active forms [1,15] (Figure 3). LOX, the isoform for which its proteolytic activation has been more clearly characterized, is synthesized as a precursor of approximately 46 kDa, which is processed to the enzymatically active form of 32 kDa. The proteolytic processing of the zymogen is facilitated by fibronectin, which binds LOX and BMP1, thereby regulating the catalytic activity of LOX [15]. It should be noted that LOX is assisted by other proteins in the cross-linking of collagen and elastin. Fibromodulin, a small leucine-rich protein (SLRP), interacts with LOX and acts as a modulator of its activity fostering a site-specific cross-linking of collagen fibrils [16]. Similarly, the interaction between LOX and fibulin-4 seems to promote elastin cross-linking [17]. Overall, LOX activity can be regulated at different levels: regulating LOX transcription, modulating the extracellular processing of the pro-enzyme, and stimulating or inhibiting the catalytic activity of the mature enzyme.

Beyond ECM cross-linking, additional biological functions have been reported for LOX and LOXLs (LOX/LOXLs) [1,2,3], including the control of cell adhesion migration and proliferation and the modulation of gene transcription and epithelial to-mesenchymal transition. Further, active intracellular forms (both cytoplasmic and nuclear) for LOX/LOXLs have been described [18,19,20], while the pro-peptide (LOX-PP) released during the proteolytic processing of LOX also exhibits biological activity [2,21] (Figure 3). Therefore, it is likely that LOX/LOXL biology continue revealing novel critical aspects in the near future.

## 3. LOX Isoenzymes in the Cardiovascular System

Early studies that associated the cardiovascular phenotype of lathyrism (characterized by aortic dissection/rupture) with the inhibition of the LOX enzymatic activity (reviewed in [22]) put the focus on the relevance of the LOX family in the cardiovascular system. Results from genetically modified animal models support a critical contribution of these enzymes to cardiovascular development, function and remodeling. LOX knockout mice (*Lox*^−/−^) develop to full term but are not viable and die at the end of the gestation or as neonates [23]. These animals show severe vascular abnormalities, most likely due to a defective elastogenesis. Tortuous aortas, a high incidence of aortic aneurysms, disruption of elastic fibers and abnormalities in the morphology and adhesive properties of endothelial and smooth muscle cells were detected in *Lox*^−/−^ pups. Surprisingly, however, *Lox*^−/−^ fetal hearts were indistinguishable from those of wild-type animals, while cardiac LOX overexpression severely impacts on cardiac function [24], as explained in more detail later in this review. Knockout mice for LOXL1, the closest mammal paralog of LOX, show an altered post-partum deposition of elastic fibers in pelvic organs associated with pelvic organ prolapse, emphysematous lungs and skin laxity, as well as vascular abnormalities, although less serious than those detected in *Lox*^−/−^ mice [25]. In turn, transgenic mice with cardiomyocyte-specific expression of LOXL1 developed cardiac hypertrophy [26]. Deletion of LOXL2 in mice increases perinatal death in nearly half of newborn animals, associated with dramatic alterations in the heart (disrupted ventricular septa) [27], while, unexpectedly, animals surviving the perinatal period grow without any major pathological manifestation. Regarding LOXL3 and LOXL4, both have been recently involved in vascular remodeling [28,29,30], although no apparent cardiovascular alterations have been detected in LOXL3 knockout mice, which exhibit perinatal lethality due to severe craniofacial defects and spinal deformities [31]. Unfortunately, LOXL4-deficient mice are not yet available.

LOX and LOXLs isoenzymes have been implicated in the control of vascular homeostasis. The expression of LOX is high in the endothelium of healthy arteries and its inhibition has been linked with the endothelial dysfunction evoked by atherosclerotic risk factors, the instability and rupture of advanced atherosclerotic plaques and aortic aneurysm development [12]. Further, LOX regulates vascular smooth muscle cells (VSMC) proliferation and vascular remodeling, participates in the control of vascular stiffness, oxidative stress and calcification, and modulates platelet activation and thrombosis [12,32,33,34]. Likewise, LOXL2 controls the age-associated increase in vascular stiffness and it is the main LOX isoenzyme contributing to neovascularization. The mechanisms underlying the LOX/LOXLs-mediated control of vascular homeostasis have been extensively discussed [12] and are beyond the scope of this review.

Regarding the heart, LOX and LOXLs are spatiotemporally regulated during embryonic and fetal heart development [35,36], and results in humans and in different experimental models have clearly evidenced that the disturbance of LOX/LOXLs expression/activity is linked to cardiovascular diseases [4,5,6,37]. Among the five LOX enzymes, LOX is the most abundant in the heart [13], and most studies on cardiac function refer to this isoenzyme, as described below. Less information is available about LOXL1 and cardiac diseases. Nonsynonymous coding single-nucleotide polymorphisms (SNPs) in the *LOXL1* gene are the major known genetic risk factor for pseudoexfoliation syndrome (XFS; OMIM#177650) [38], an aging-related systemic disease involving an abnormal ECM deposition, characterized by an increased risk of glaucoma, and a high susceptibility to heart disease among others [39]. In a model that spontaneously develops age-related cardiac-selective fibrosis, the plasminogen activator inhibitor-1 (PAI-1) knockout mice, genome-wide gene expression profiling identified *Loxl1* among the most upregulated transcripts involved in profibrotic pathways [40]. Concerning LOXL2, it is highly expressed during the early stages of cardiac development [13], has been recognized as a NOTCH candidate gene potentially involved in valve formation [41], and is a major player in cardiac fibrosis [42]. The contribution of each member of the LOX family to cardiac diseases has been more exhaustively detailed in the next sections.

## 4. ECM Synthesis and Remodeling in the Heart

In the adult mammalian heart, cardiomyocytes are arranged in layers separated by clefts. An intricate network of ECM proteins provides a scaffold for the cellular components and participates in the transmission of the contractile force. Cardiac ECM is mainly composed of fibrillar type I and III collagens (approximately 85% and 11% of total myocardial collagen, respectively), and minor components including elastin, laminin, and fibronectin [43]. Cardiac ECM also contains latent growth factors and proteases whose activation, following cardiac injury, triggers fibrosis, an anomalous matrix remodeling due to an disproportionate deposition of ECM proteins during the wound healing response associated with chemical, mechanical, and immunological stresses.

Mature fibrillar collagen is highly stable (half-life 80–120 days), and its turnover is primarily regulated by cardiac fibroblasts. The homeostatic control of cardiac ECM involves a tightly regulated balance between synthesis and degradation of matrix proteins, whose disturbance results in structural and functional abnormalities of the heart. Collagens are synthesized as procollagen chains, with N- and C-terminal propeptide domains, which associate into trimers allowing the folding of the collagen triple helix [44]. Procollagen molecules translocate to the Golgi apparatus, where they are packaged in vesicles for their extracellular transport. Once the terminal propeptides are cleaved by zinc (Zn)-dependent metalloproteinases (procollagen N- and C-proteinases), mature collagen molecules spontaneously self-assemble into fibrils [45]. Fibrillar type I and III collagens must be cross-linked to form fibers highly resistant to proteolytic degradation and to acquire their physical properties and tensile strength. Collagen experiences two main types of cross-links: those mediated by LOX enzymes and those derived from the nonenzymatic glycation of lysine and hydroxyllysine residues [46].

Besides cardiomyocytes, fibroblasts represent the largest cardiac cell population. Cardiac fibroblasts, as the main ECM-producing cells, play a key role in preserving the integrity of the ECM network. The cardiac fibroblast population significantly expands during the two first weeks after birth due to the increase of left ventricular pressures [47]. In healthy young adult hearts, cardiac fibroblasts remain quiescent and secrete negligible amounts of ECM proteins [48]. Following cardiac injury, however, a differential population of fibroblasts, namely myofibroblasts, accumulates in sites of injury. Myofibroblasts behave as phenotypically modulated cardiac fibroblasts which combine ultrastructural and phenotypic characteristics of smooth muscle cells (contractile stress fibers) and synthetically active fibroblasts (extensive endoplasmic reticulum) [48]. Several cues, including an abnormal composition and mechanical characteristics of the ECM, upregulation and release of cytokines and growth factors, and enhanced mechanical stress, govern myofibroblast transdifferentiation. Collagen mainly originates from activated myofibroblasts which play a fundamental role in both reparative and fibrotic processes. Myofibroblast transdifferentiation and enhanced deposition of fibrillar collagen in the cardiac interstitium are characteristic of the cardiac fibrotic response.

Fibrillar collagens of the ECM form a complex meshwork that provides structural and biochemical support to cardiac cells and regulates cell signaling. Cardiac function is unequivocally influenced by the composition and structure of the ECM and any disturbance of the ECM contribute to the progression of cardiac diseases. Firstly, in response to injury, collagen deposition and ECM remodeling are adaptive responses that aim to preserve tissue integrity and cardiac function. However, the synergistic response to proinflammatory and profibrotic mediators may overactivate myofibroblasts, leading to an excess of collagen deposition in the matrix. Irrespective of the nature of the injury, and whether it is focal (e.g., MI) or more global (e.g., pressure or volume overload), the common fibrotic response is characterized by collagen synthesis and deposition and upregulation of LOX enzymes, leading to a consequent increase in CCL which makes fibrillar collagen less prone to degradation by matrix metalloproteinases (MMPs). This process alters ECM biomechanical properties and thereby, determines diastolic dysfunction and cardiac stiffness [4,7,37].

Essentially, fibrosis is characteristic of all forms of heart disease, being detrimental for LV function and contributing to the progression to HF. Most of the pathophysiological stimuli that trigger cardiac fibrosis modulate LOX/LOXLs that play a critical role in the process. It is well known the importance of transforming growth factor beta-1 (TGF-β1) in cardiac remodeling and fibrosis [49,50]. LOX/LOXLs are regulated by TGF-β in multiple cells and tissues including the heart. The induction of LOX by TGF-β1 in cardiac fibroblasts requires the activation of PI3K/Akt, Smad3, and MAPK signaling. Concomitantly TGF-β1 increases collagen types I and III, and BMP1 expression [8], thereby contributing to the fibrotic response. Similarly, the induction of LOX by TGF-β1/Smad/AP-1 signaling exacerbates myocardial fibrosis and HF induced by abdominal aortic coarctation in rats [9]. Other profibrotic mediators, including pro-inflammatory cytokines such as TNFα are upregulated in many cardiac diseases. TNFα is a fundamental factor regulating heart function and cardiac fibrosis in a LOX dependent manner. In fact, this cytokine increases LOX expression in cardiac fibroblasts through TGF-β and PI3Kinase signaling pathways [10]. As reviewed below, the increase in LOX/LOXLs expression has been linked with the detrimental cardiac remodeling and dysfunction in several pathological scenarios.

### 4.1. LOX and LOXLs in Dilated and Hypertrophic Cardiomyopathies and HF

The increase in LOX/LOXL expression and/or LOX activity seems to be required by the cardiac fibrotic process to progress. The critical role of the LOX family in CCL determines the close link between LOX expression and/or activity and both myocardial stiffness and disturbed LV function. Clinical data and findings from multiple studies in animal models support this relationship.

LOX has been involved in the fibrotic process that accounts for end-stage dilated cardiomyopathy (DCM). The increase of LOX mRNA and protein levels in the myocardium in patients with DCM and end-stage HF were associated with a rise in both TGF-β expression and collagen content [51]. Similarly, LOX is increased in areas of intersticial and perivascular fibrosis in the fibrotic myocardium of patients with hypertensive heart disease (HHD) and chronic HF [6]. In these patients a positive correlation between LOX protein levels and CCL, and between CCL and LV stiffness were found. Subsequent studies further support the concept that the degree of CCL (rather than the total amount of collagen) correlates with an impairment of systolic and diastolic function. Patients with HF and normal ejection fraction (EF), displayed an increase in the myocardial content of collagen type I, enhanced CCL, and higher LOX expression, which were associated with a deterioration of diastolic function [7]. Likewise, in patients with hypertension and stage C heart failure, CCL, and not collagen amount, was associated with an elevation of filling pressures [37]. This study concludes that the disproportionate CCL promoted an increase in LV stiffness, which leads to an elevation of filling pressures in these patients [37]. In agreement, in HF patients with normal EF, Kasner et al. found an enhanced myocardial collagen content, but also an increase in LOX expression and CCL, both associated with an abnormal diastolic function [7]. Remarkably, patients with HHD and HF, exhibited an excessive myocardial expression of osteopontin (OPN), that was related to enhanced LOX, higher insoluble collagen, as well as LV stiffness and altered systolic function. Since OPN up-regulates LOX in human fibroblasts in culture, the authors proposed that the OPN-LOX axis might promote an exacerbated deposition of insoluble collagen (stiffer and more resistant to degradation) and the consequent alteration of LV function in these patients [52]. The increase of LOX and OPN levels, as well as those of TGF-β1 and periostin, in cardiac tissues from patients with end-stage HF has been confirmed in subsequent studies [53]. Interestingly, recent research shows the relevance of LOX as a target of miRNAs that regulate cardiac function and fibrosis, in particular of miR-19b [54]. In patients with severe aortic stenosis (AS) and HF, cardiac and circulating miR-19b levels were inversely correlated with LOX protein levels, CCL and LV stiffness [54]. Concerning LOXLs and similar to LOX, cardiac LOXL2 is upregulated in patients with ischemic or idiopathic DCM, in which LOXL2 levels correlate with CCL and with diastolic dysfunction [42]. Furthermore, circulating LOXL2 levels are also elevated in HF patients and correlate with other biomarkers of HF [42]. Therefore, both LOX and LOXL2 contribute to cardiac CCL and condition cardiac performance.

Experimental research in different animal models of cardiac hypertrophy has also contributed to build the concept that qualitative rather that quantitative changes in collagen critically impact on cardiac stiffness and remodeling [5]. Certainly, multiple studies have evidenced enhanced expression and/or activity of LOX/LOXLs and CCL and their relationship with adverse myocardial remodeling and dysfunction. Myocardial TGFβ-dependent signaling, LOX expression and CCL were found increased in spontaneously hypertensive rats (SHRs) with LV hypertrophy compared with normotensive animals [55]. Further, in BALB/c mice subjected to experimental hypertension induced by N(G)-nitro-l-arginine methyl ester (l-NAME), the cardiac increase in total fibrillar collagen, CCL, and in the expression and enzymatic activity of LOXL-3 were associated with ventricular stiffness [56]. A similar situation was reported in a model of chronic HF induced by aortic banding, in which the TGF-β1/Smad/AP-1-dependent induction of LOX aggravates myocardial fibrosis and HF [9]. The enhanced LOX-dependent CCL also underlies the pro-hypertrophic role of syndecan 4 in the pressure-overloaded myocardium. The cardiac expression of syndecan 4 rises in mice subjected to aortic banding and impacts on LOX and CCL through a dual mechanism. On one hand, the cytosolic domain of syndecan 4 is responsible for a NFAT-mediated induction of collagen, OPN, and LOX, while its extracellular domain additionally facilitates LOX-dependent CCL by interacting with collagen fibers [57].

In this scenario, disrupting CCL through inhibition of LOX is expected to reverse fibrosis and ameliorate cardiac dysfunction. Indeed, the administration of β-aminopropionitrile (BAPN), an irreversible LOX inhibitor, to 38-week-old mice reversed established age-related myocardial fibrosis to a level similar to that of young mice [58]. The inhibition of LOX by BAPN modulated TGF-β signaling and collagen synthesis, but also the degree of macrophage infiltration [58]. In the rat model of aortocaval fistula-induced volume overload (VO), the expression and activity of LOX, as well as collagen and CCL, were progressively upregulated in parallel with an enhanced ventricular mass [59]. Interestingly, in this model, LOX activity negatively correlated with cardiac function, while LOX inhibition by BAPN completely circumvented the increase in fibrotic mediators, such as collagens, MMPs and their tissue inhibitors, and ameliorated the cardiac dysfunction caused by VO [60,61].

**Figure 4:**
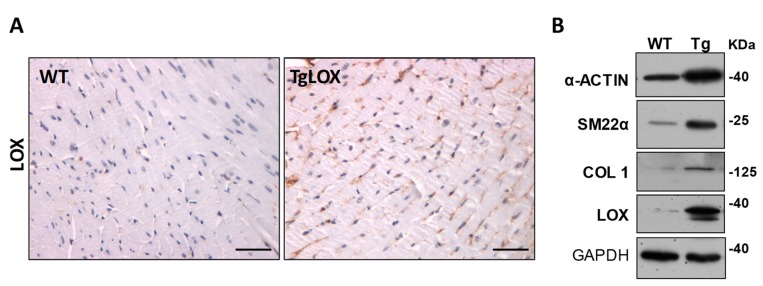
Impact of LOX transgenesis on cardiac fibroblast phenotype. (**A**) LOX immunohistochemical analysis in heart sections from WT and TgLOX mice evidences the increased cardiac LOX expression in LOX transgenic mice. Bar: 50 µm. (**B**) In cardiac fibroblasts from TgLOX mice, which exhibits higher LOX expression than WT cells, the expression of markers of the phenotypic switch of fibroblasts to myofibroblasts (α-actin and transgelin; SM22α), as well as that of type I collagen, were increased. Representative images of Western-blot assays are shown. Glyceraldehyde 3-phosphate dehydrogenase (GAPDH) protein levels were analyzed as loading control in Western-blot assays. The position of the molecular weight standards (HyperPAGE prestained protein markers, BIOLINE; Ref: BIO-33065) is indicated.

**Figure 5:**
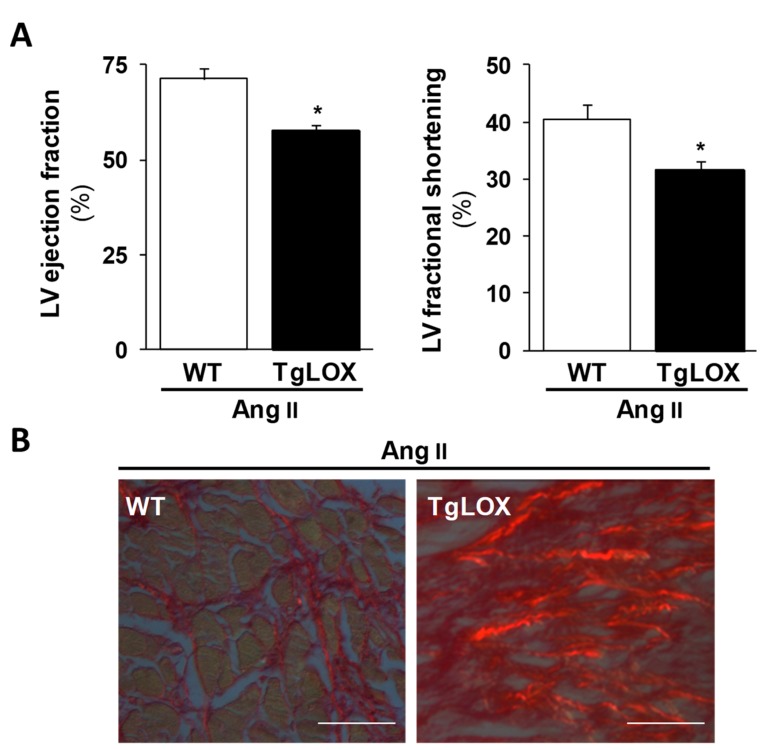
LOX transgenesis exacerbates Ang II-induced LV systolic dysfunction and fibrosis. (**A**) WT (white columns) and TgLOX (black colums) mice were infused with AngII (1000 ng/kg/min) or saline solution for 14 days. The overexpression of LOX reduces left ventricular (LV) ejection fraction (left panel) and fractional shortening (right panel) as determined by echocardiography in Ang II infused mice. Results are expressed as mean ± SD. * *p* < 0.05 vs. WT. (**B**) Heart sections from Ang II-infused mice were stained with Picrosirius red. Under polarized light, cross-linked collagen appears as red refringent fibers. As shown, LOX overexpression enhances the Ang II-induced deposition of mature collagen (Bar: 50 µm).

Therefore, LOX inhibition seems to be cardioprotective, while the chronic increase of cardiac LOX activity would play a detrimental role favoring the transition from compensated remodeling to decompensated failure aggravating HF. Our recent study, using a new transgenic mouse model (TgLOX) that overexpresses human LOX in the heart (Figure 4A), further evidences the direct relationship between LOX and diastolic function [24]. Myocardial LOX overexpression causes an age-dependent diastolic dysfunction and accelerates cardiac remodeling [24]. More interestingly, LOX transgenesis exacerbated the cardiac hypertrophy and dysfunction induced by the chronic infusion of Angiotensin II (Ang II) (Figure 5A), triggering a more severe fibrotic process with higher collagen deposition and CCL (Figure 5B) and an enhanced expression of fibrotic markers.

**Figure 6:**
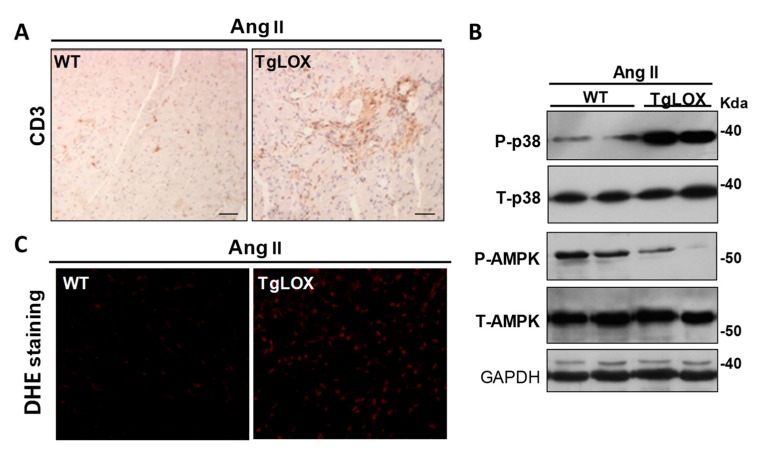
LOX transgenesis aggravates Ang II-induced cardiac inflammation and oxidative stress and disturbs Ang II-dependent signaling pathways. (**A**) WT and TgLOX mice were infused with AngII (1000 ng/kg/min) or saline solution for 14 days. Inmunohistochemistry revealed that Ang II-challenged TgLOX mice exhibited higher myocardial lymphocyte infiltration (CD3+) than WT animals. (**B**) Representative Western blot assays performed in heart lysates from these animals showing that LOX transgenesis alters the activation by phosphorylation of both p38 MAPK (p38) and AMPK (p-p38 and p-AMPK, respectively). Total p38 and AMPK levels are also shown. Glyceraldehyde 3-phosphate dehydrogenase (GAPDH) protein levels were analyzed as loading control. The position of the molecular weight standards (HyperPAGE prestained protein markers, BIOLINE; Ref: BIO-33065) is indicated. (**C**) Dihydroethidium (DHE) staining revealed that LOX overexpression exacerbates the Ang II-mediated increase in cardiac superoxide anion production.

**Figure 7:**
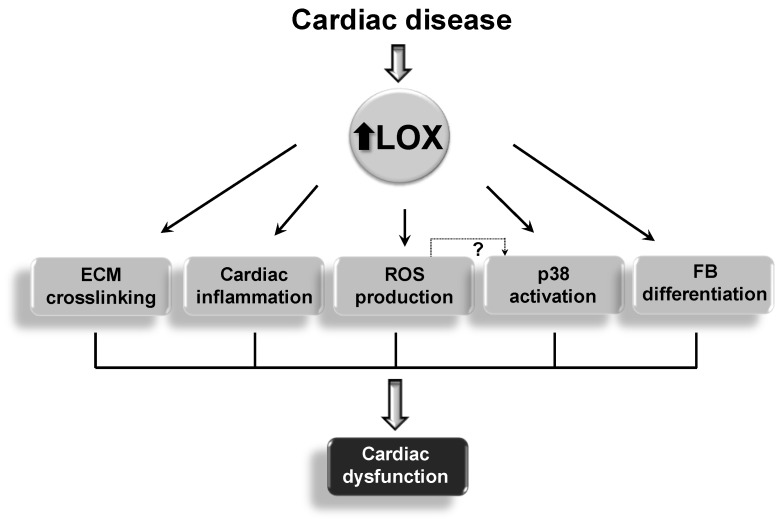
The upregulation of LOX/LOXLs in cardiac diseases affects several processes that contribute to cardiac dysfunction. ECM: extracellular matrix; FB: fibroblast; ROS: reactive oxygen species.

Additionally, LOX transgenesis exacerbated the cardiac inflammatory response induced by Ang II (Figure 6A), enhanced the expression of proinflammatory markers such as *Il-6*, and decreased that of the cardioprotective factor *Fgf21* [24]. These animals also exhibit an altered Ang II-dependent signaling with increased p38 MAPK activation, while reduced AMP-activated protein kinase (AMPK) activation (Figure 6B). Further, LOX enhances cardiac oxidative stress (Figure 6C), an effect due, at least in a part, to the attenuation of the induction of SOD1 promoted by Ang II. Whether the enhanced cardiac oxidative stress exhibited by TgLOX mice accounts for the overactivation of p38 MAPK, as described in the vascular wall in VSMC-specific LOX transgenic mice [33], is an issue that deserves further investigation. Therefore, beyond CCL other mechanisms including excessive inflammation and ROS production could account for the cardiac phenotype exhibited by TgLOX mice (Figure 7). Further, this study reveals that LOX promotes fibroblast-to-myofibroblast transition. Indeed, transgenic cardiac fibroblasts expressed higher levels of markers of the phenotypic switch of fibroblasts to myofibroblasts (i.e., α-alpha smooth muscle actin and transgelin [SM22α]) than control cells (Figure 4B).

Similarly, Ang II-induced cardiac hypertrophy was exacerbated in transgenic mice that overexpress LOXL1 specifically in cardiomyocytes, which exhibited myocyte hypertrophy, and higher mRNA levels of brain natriuretic peptide (Bnp) than their WT littermates. Echocardiographic data evidenced that LOXL1 transgenesis caused an increase in wall thickness with preserved cardiac contraction [26]. It should be noted that *Loxl1* expression is induced by hypertrophic agonists in cardiomyocytes in culture, and, in vivo, in a rat model of hypertrophy induced by abdominal aortic constriction. Thus, LOXL1 also seems to critically contribute to cardiac hypertrophy. Finally, and consistent with data from HF patients described above, LOXL2 seems to be fundamental for interstitial fibrosis and cardiac dysfunction. In mice, pressure overload activates fibroblasts and induces the expression of LOXL2, leading to fibrosis and disturbing systolic and diastolic functions [42]. Inhibition of LOXL2 by antibody-based strategies or gene knockdown limits cardiac fibrosis and LV dilatation, ameliorating systolic and diastolic dysfunction. LOXL2 stimulate myofibroblast migration acting downstream of TGF-β2 and through a PI3K/AKT mediated mechanism that promotes fibroblast-to-myofibroblast transformation [42]. Therefore, both LOX and LOXL2 contribute to the phenotypic switch of fibroblasts emphasizing the importance of ECM stiffness in myofibroblast activation [62].

### 4.2. LOX/LOXLs in Post-MI Cardiac Remodeling

The human heart has a negligible capacity to cardiomyocyte regeneration after MI. MI is followed by an extensive remodeling of the ECM characterized by an important accumulation of collagen that triggers both replacement and interstitial fibrosis and scar formation. Post-MI fibrosis, developed in both infarcted and non-infarcted cardiac regions, is essential for the recovery of heart function.

The cellular response to infarction can be divided into three overlapping phases: the inflammatory phase of infarct healing, the proliferative phase of cardiac repair, and the formation of a mature scar [43]. During the inflammatory phase an enhanced protease activity leads to an extensive degradation of the cardiac ECM, and to the production of a provisional fibrin and fibronectin matrix that acts as a scaffold for inflammatory cells. The proliferative phase is featured by the transdifferentiation of myofibroblasts and the deposition of fibrous tissue. Cardiac fibroblasts play a critical role in LV healing and remodeling after MI. They are reprogrammed into myofibroblasts that are instrumental for the formation of contractile and mature collagen scars that limit the detrimental dilation of infarcted areas. Finally, during the formation of a mature scar, increased LOX promotes the formation and deposition of stiff collagen fibers [63]. Thus, cardiac fibrosis leads to increased collagen deposition and cardiac stiffness derived from an exacerbated CCL. Although the fibrotic response aims to preserve tissue integrity, progressively this pathological fibrosis disturbs LV function linked to poor prognosis associated with a marked increase in the risk of HF, ventricular arrhythmias, and sudden cardiac death.

Early studies reported increased collagen content and CCL in the viable free wall of rats subjected to surgery to produce moderate-to-large transmural infarcts [64]. This research suggests that LOX-mediated CCL also determines the adverse remodeling occurring in remote zones to MI, which contributes to HF of ischemic origin. The concomitant post-MI upregulation of LOX and CCL has been demonstrated in multiple animal models. In a rhesus monkey model of MI, ischemic injury generated by ligation of the left anterior descending (LAD) artery promoted an increase in the collagen type I/III ratio in the scar area. Coincidently, LOX expression was upregulated in both the scar area and the border zone, while the expression of MMP1 was downregulated [65]. The degree of LOX upregulation and fibrosis after MI has been related with the extent of LV dysfunction. Indeed, LV fibrosis and higher mRNA levels of collagen I, connective tissue growth factor (Ctgf), Tgfβ and Lox were observed in rats subjected to LAD coronary artery ligation presenting signs of HF versus non-failing hearts [66]). Similarly, in mice, MI upregulated the expression of LOX and LOXL1-4 promotes the accumulation of mature collagen fibers in the infarcted area [67]. Both TGFβ and hypoxia-dependent signaling pathways underlie the upregulation of LOX/LOXLs in cardiac fibroblasts, in particular in areas of extensive remodeling. In this model, collagen accumulation and CCL maturation were ameliorated by a post-infarction treatment with BAPN or with a neutralizing antibody against LOX, which also reduced ventricular dilatation and improved cardiac function [67]. It should be noted, however, that a severe impairment of LOX activity and CCL might result in defective scar collagen maturation, compromised scar contractility, and exacerbated adverse remodeling. Indeed, in AMP-activated protein kinase (AMPKα1) knockout (KO) mice subjected to permanent LAD ligation, a reduction in scar maturation and contractility evoked an exaggerated LV dilation. AMPKα1 knockdown was associated with an enhanced proliferation of cardiac fibroblasts in infarcted areas, a reduced expression of α-SMA, a marker of fibroblast transdifferentiation, and a defective maturation of myofibroblasts. These effects might be secondary to the strong down-regulation of the non-canonical TGF-β1/p38 MAPK pathway, and the dramatic reduction of LOX protein levels in the scar of KO hearts [68]. α2β1 integrin has also been involved in the modulation of LOX after MI through its interaction with collagen I [69].

### 4.3. LOX Activity in Cardiac Remodeling and Fibrosis Associated With Obesity and Metabolic Syndrome

HFPEF is a common clinical syndrome with poor prognosis strongly associated with obesity and the metabolic syndrome. HFPEF is characterized by diastolic dysfunction, or reduced capacity of the heart to fill during diastole, associated with high LV end diastolic blood pressure. The etiology of diastolic dysfunction is multifactorial and includes abnormalities in cardiac ECM remodeling and fibrosis. The key role of LOX in cardiac function and remodeling in this pathophysiological scenario is documented in models of diet-induced cardiac dysfunction. In a murine model of diet-induced metabolic syndrome (high-fat high-simple carbohydrate, HFHSC) no changes were observed in the cardiac expression of *Lox* and *Loxl3*; however, metabolic syndrome significantly upregulated *Bmp1*, which increased LOX processing and activity and CCL [70]. The LOX-mediated fibrotic response is concurrent with a marked rise in end-diastolic pressure, higher LV stiffness, and abnormal diastolic filling pattern [70]. Alterations in cardiac ECM CCL seem to be the underlying cause of diastolic dysfunction, and it has been proposed a regulatory role for the adaptive immune system. Indeed, results in mice devoid of functional T lymphocytes fed a HFHSC diet, suggest a role for these inflammatory cells in the modulation of LOX-dependent collagen maturation [71]. Treatment of mice with selective TH1 lymphocyte inducers revealed TH1 immune polarization associated with an increase in cardiac *Lox* and *Loxl3* expression, LOX activity, CCL and ventricular stiffness and a concurrent decrease in cardiac output [72]. Therefore, TH1 lymphocytes are involved in of the development of CCL-mediated diastolic dysfunction, which has potential clinical application for the treatment of diastolic HF [72].

In a recent study we analyzed whether inhibition of LOX activity impacts on the cardiovascular remodeling of ECM triggered by obesity [73]. We found that cardiac LOX was upregulated in rats fed a high-fat diet (HFD), together with cardiac and vascular fibrosis and higher levels of superoxide anion (O_2_^−^), collagen I and TGF-β. LOX inhibition by BAPN significantly attenuated these changes and improved cardiac hypertrophy. Interestingly, BAPN also ameliorated the HFD-dependent increase in circulating leptin levels. In turn, in cardiac fibroblasts, leptin enhanced collagen I, TGF-β and CTGF levels, Akt phosphorylation and O_2_^−^ production, while LOX inhibition attenuated these responses. Therefore, LOX inhibition limited the HFD-mediated fibrosis and oxidative stress in the cardiovascular system. The ability of BAPN to reduce leptin levels in vivo and prevent the leptin-mediated induction of profibrotic mediators and ROS in cardiac cells suggests that the interaction between leptin and LOX regulates downstream signaling pathways underlying the development of myocardial fibrosis in obesity.

Finally, LOX has been related to the pro-fibrotic effects and cardiac stiffening caused by the intake of high n-6 polyunsaturated fatty acid (PUFA) diets. In cardiac fibroblasts in culture, high concentrations of linoleic acid (mimicking an excess of dietary n-6 PUFA) upregulated collagen I and LOX [74].

### 4.4. LOX and LOXL2 in Atrial Fibrillation

Atrial fibrillation (AF) is commonly characterized by increased atrial fibrosis and structural remodeling. Enhanced LOX expression and CCL underlies interstitial fibrosis and remodeling in AF. Indeed, upregulation of LOX has been documented in AF both in humans and in animal experimental models [75,76,77,78]. The left atrial appendage from patients with AF displayed an increase in collagen content and cross-linking, as well as enhanced expression of LOX, CTGF and fibronectin and higher Rac1 activity [76]. The mechanism underlying these effects was deciphered using pharmacological and gene knockdown strategies in both cardiac fibroblasts and transgenic mice with cardiac overexpression of constitutively active Rac1 (RacET), which develop atrial fibrosis and spontaneous AF with aging, characterized by increased LOX expression, collagen content, and cross-linking. These studies evidence that Ang II enhances the expression of LOX and fibronectin through the activation of Rac1 GTPase and CTGF. Notably, in RacET mice, torasemide, reduced the aldosterone synthase-mediated profibrotic signaling limiting the induction of CTGF and LOX, preventing atrial fibrosis and reducing the prevalence of AF [77]. Therefore, LOX is involved in a signaling pathway that represents a target for the prevention of fibrotic atrial remodeling.

Little information is available about the involvement of other LOX isoenzymes in AF. Recent evidence indicates that serum LOXL2 levels were augmented and correlated with the level of left atrial fibrosis [79]. Indeed, higher LOXL2 levels were found in AF patients with a size of left atrium (LAD) ≥ 40 mm compared with those with LAD < 40 mm, and multivariate regression analysis revealed that they were independent predictors of AF [79]. These data suggest that LOXL2 levels may predict the degree of atrial fibrosis in AF patients and support the contribution of this isoenzyme to the pathogenesis of AF.

### 4.5. LOX in Other Pathophysiological Settings Leading to Cardiac Dysfunction

In addition to the situations already mentioned, LOX is modulated by a wide array of conditions that impair cardiac performance. This has been evidenced in a variety of animal experimental models. In dystrophin-deficient mice, a model of Duchenne muscular dystrophy (DMD) which develops fibrosis and DCM, an altered profibrotic gene expression profile, in particular, a steep increase in *Lox* mRNA expression has been documented [80]). The renin–angiotensin–aldosterone system, one of the pathways involved in adverse cardiac remodeling, has been linked to a disturbance in *Lox* expression. In particular, experiments in null mice evidence that the control of myocardial *Lox* expression depends on TRAF3 Interacting Protein 2 (TRAF3IP2), a redox-sensitive adaptor molecule and a decisive signaling intermediate in aldosterone/salt-induced cardiac hypertrophy and fibrosis [81]. Likewise, in rats, the hypertrophic and profibrotic cardiac effects of high molecular weight advanced glycation end products (HMW-AGEs) were likely due to the increase of LOX expression and the LOX-mediated CCL, which might be responsible for the higher cardiac stiffness promoted by these compounds [82]. Arrhythmogenic right ventricular cardiomyopathy (ARVC) is a severe disease featured by an extensive fibrotic replacement. In animal models that recapitulate human ARVC, the cardiac fibrotic response was associated with an upregulation of *Lox* [83,84]. Finally, a strong induction of LOX was identified as the underlying cause of the restrictive cardiomyopathy evoked by a sustained running in SHR rats. The upregulation of LOX results in the deposition of an extensive network of matrix proteins and a substantial elevation of collagen III expression, thereby contributing to the filling disorder in trained SHR [85].

## 5. LOX/LOXLs as Pharmacological Targets in Myocardial Diseases

The strong impact of the disturbance of LOX/LOXLs activity on CCL and heart performance [6,7,37] supports the interest of LOX/LOXLs-inhibitory therapies to improve cardiac remodeling and slow HF. Moreover, and considering that tissue drug uptake and distribution could be impaired by an excessive LOX-mediated ECM deposition [86], interfering with cardiac collagen stabilization by LOX/LOXLs inhibition could additionally improve the response to specific pharmacological therapies.

Despite the potential interest of LOX inhibitors, LOX-targeting drug discovery has been hampered by the lack of LOX/LOXLs crystallographic structure, an area of active investigation, as indicate recent promising data aiming to clarify the structural–functional relationship of LOXL2 [87]. Furthermore, safety concerns should be considered, since increased expression/activity of these isoenzymes has been associated with cardiac diseases, but a decrease of LOX could underlie the development of several vascular pathologies and promote plaque instability [12]. Because cardiac and vascular pathologies frequently coexist, therapeutic approaches aiming LOX inhibition must be thoroughly evaluated to exclude undesirable side-effects. Moreover, chronic and irreversible cardiac LOX inhibition might cause a severe disruption of the ECM and compromise the structural integrity of the heart. Overall, these considerations evidence that irreversible inhibitors of LOX/LOXLs have critical concerns as therapeutic tools for chronic diseases. In fact, the chronic administration of the broadband LOX inhibitor BAPN provokes neurotoxicity and lathyrism, a disorder characterized by serious connective tissue defects which severely disturbs bone mechanical strength and blood vessel mechanics [22].

Given these constraints, alternative approaches have been assessed (Table 1). Monoclonal antibodies against LOX and LOXL2 have demonstrated their ability to ameliorate cardiac dysfunction and fibrosis in response to pressure overload or MI in mice [42,67]. Simtuzumab, a humanized monoclonal antibody against LOXL2, developed as anti-fibrotic agent, emerges as an interesting therapeutic tool for cardiac diseases. However, and despite promising preclinical data, this drug showed no significant clinical benefit in reducing liver and lung fibrosis [88], and clinical trials testing its efficacy against cardiac fibrosis and dysfunction have not been implemented. Besides monoclonal antibodies, efforts have been made for developing small-molecules that directly inhibit LOXL2 catalytic activity [89]. PXS-5153A, a novel dual LOXL2/LOXL3 inhibitor, dose-dependently decreased LOXL2-mediated collagen oxidation and CCL in vitro, and improved cardiac output after MI in mice [90].

Alternative approaches which could achieve an effective reduction of LOX activity might be derived from the use of metal chelators, which would reduce copper availability and therefore impair LTQ biogenesis, or the blockade of LOX and LOX1 extracellular processing by inhibiting BMP1.

It should be highlighted, that the benefit of some drugs widely used for the management of cardiovascular diseases could be derived, at least in part, by their ability to inhibit LOX. This is particularly the case of torasemide, which reduces myocardial LOX expression, CCL and fibrosis and normalizes LV stiffness in patients with hypertensive HF [6]. Similar effects could be ascribed to the losartan metabolite EXP3179 in hypertensive rats [91]). In fact, despite EXP3179 exerted lower antihypertensive effects than EXP3174, a second losartan metabolite that neither modified LOX nor CCL, EXP3179, showed higher anti-fibrotic efficacy than EXP3174.

LOX/LOXLs expression and/or activity can also be modulated by molecules interfering mechanisms and signaling pathways involved in cardiac remodeling. This has been illustrated in different experimental models. In SHR, which showed a significant increase cardiac LOX and CCL accompanied by a rise in phosphorylated Smad2, CTGF, and collagen type I, a synthetic peptide from TGF-β1 type III receptor (P144) limits myocardial fibrosis [55]. In rats infused with Ang II, the tetrapeptide Ac-SDKP (*N*-acetyl-seryl-aspartyl-lysyl-proline), prevented the Ang II-induced increases in LOX and LOXL1 levels, collagen and CCL, TGFβ signaling, NF-κB activation, and the infiltration of CD4+/CD8+ lymphocytes and CD68+ macrophages [92]. In turn, finerenone (a non-steroidal mineralocorticoid receptor antagonist) decreased the expression of CTGF and prevented the aldosterone-induced upregulation of CTGF and LOX. Finerenone attenuated the Rac1 GTPase-mediated upregulation of TGF-β, and in RacET mice, which exhibit increased LV end-diastolic and end-systolic volumes and high TGF-β, CTGF and LOX levels, this drug prevented LV dilatation [93]. Finally, the strong anti-fibrotic potential of the pharmacological inhibition of ROCK is in part due to a decrease in the expression of LOX. Indeed, in a model of engineered connective tissue, composed by cardiac fibroblasts and collagen, Rho-associated kinases (ROCK1 and ROCK2) were identified as key mediators of TGF-β-dependent tissue stiffening of engineered heart muscle tissue, and LOX as a downstream target of this ROCK-actin-MRTF/SRF pro-fibrotic signaling pathway [94].

## 6. Conclusions and Therapeutic Opportunities

The findings summarized in this review evidence the critical contribution of the LOX family to cardiac function and remodeling. The upregulation of LOX/LOXLs seems to be a feature of several fibrotic processes affecting the heart, from those associated to post-MI cardiac remodeling, cardiac hypertrophy triggered by pressure or volume overload, HFPEF associated with obesity and metabolic syndrome or AF. Because LOX/LOXLs play a critical role in CCL, LOX activity emerges as a key biological point to control the quality of ECM, which determines the progression and severity of those clinical entities that course with fibrosis. Concerning therapeutics and although much progress has been made in the search of new antifibrotic agents useful for the treatment of cardiac fibrosis and dysfunction, clinical trials assessing the effectiveness of a priori potentially applicable drugs have failed. Therefore, further efforts will be required to better understand of LOX/LOXLs biology. LOX and LOXLs are also regarded as promising tools for new approaches to grafting and tissue regeneration, but their role in cardiac repair and tissue engineering should also be investigated in more detail.

## Figures and Tables

**Table 1:** Therapeutic approaches targeting LOX and LOXLs.

Therapeutic Approach	Experimental Model	Effect on Cardiac Remodeling and Function	Author/Year/Reference ^#^
BAPN	Mouse model of age-related myocardial fibrosis	Reduced myocardial fibrosis	Rosin et al. 2015 [58]
BAPN	Rat model of cardiac fibrosis induced by HFD	Reduced fibrosis and improved cardiac remodeling	Martínez–Martínez et al. 2016 [73]
BAPN or Anti-LOX antibody	Mouse model of MI (LAD coronary artery ligation)	Reduced ventricular dilatation and improved cardiac function	González–Santamaría et al. 2016 [67]
Anti-LOXL2 antibody	Mouse model of pressure overload (Transaortic constriction)	Reduced cardiac fibrosis and chamber dilatation, improved systolic and diastolic functions	Yang et al. 2016 [42]
BAPN	Rat model of aortocaval fistula-induced volume overload	Reduced myocardial fibrosis and improved LV stiffness and cardiac function	El Hajj et al. 2016 [60] El Hajj et al. 2018 [61]
PXS-5153A (LOXL2/LOXL3 inhibitor)	Mouse model of MI (LAD coronary artery ligation)	Reduced fibrosis and improved FS and EF	Schilter et al. 2019 [90]

Abbreviations: BAPN, β-aminopropionitrile (a pan-LOX inhibitor); EF, ejection fraction; FS, fractional shortening; HFD, high fat diet; LAD, left anterior descending; LV, left ventricular; MI, myocardial infarction.
